# Cross Deep Learning Method for Effectively Detecting the Propagation of IoT Botnet

**DOI:** 10.3390/s22103895

**Published:** 2022-05-20

**Authors:** Majda Wazzan, Daniyal Algazzawi, Aiiad Albeshri, Syed Hasan, Osama Rabie, Muhammad Zubair Asghar

**Affiliations:** 1 Computer Science Department, Faculty of Computing and Information Technology, King Abdulaziz University, Jeddah 21589, Saudi Arabia; aaalbeshri@kau.edu.sa; 2 Information Systems Department, Faculty of Computing and Information Technology, King Abdulaziz University, Jeddah 21589, Saudi Arabia; dghazzawi@kau.edu.sa (D.A.); shhassan@kau.edu.sa (S.H.); obrabie@kau.edu.sa (O.R.); 3 Institute of Computing and Information Technology (ICIT), Gomal University, Dera Ismail Khan 29050, Pakistan; zubair@gu.edu.pk

**Keywords:** internet of things (IoT), IoT malware, IoT botnet, IoT botnet detection, anomaly detection, machine learning, deep learning, kill chain model, Mitre

## Abstract

In recent times, organisations in a variety of businesses, such as healthcare, education, and others, have been using the Internet of Things (IoT) to produce more competent and improved services. The widespread use of IoT devices makes our lives easier. On the other hand, the IoT devices that we use suffer vulnerabilities that may impact our lives. These unsafe devices accelerate and ease cybersecurity attacks, specifically when using a botnet. Moreover, restrictions on IoT device resources, such as limitations in power consumption and the central processing unit and memory, intensify this issue because they limit the security techniques that can be used to protect IoT devices. Fortunately, botnets go through different stages before they can start attacks, and they can be detected in the early stage. This research paper proposes a framework focusing on detecting an IoT botnet in the early stage. An empirical experiment was conducted to investigate the behaviour of the early stage of the botnet, and then a baseline machine learning model was implemented for early detection. Furthermore, the authors developed an effective detection method, namely, Cross CNN_LSTM, to detect the IoT botnet based on using fusion deep learning models of a convolutional neural network (CNN) and long short-term memory (LSTM). According to the conducted experiments, the results show that the suggested model is accurate and outperforms some of the state-of-the-art methods, and it achieves 99.7 accuracy. Finally, the authors developed a kill chain model to prevent IoT botnet attacks in the early stage.

## 1. Introduction

The Internet of Things (IoT) collects and monitors abundant data through connected devices, thus allowing an infinite number of functions that serve the current era with its various innovations that depend on data processing. Researchers expect that, by 2024, the number of IoT links will reach 83 billion [1], which reflects the exponential growth of Internet of Things devices that impact our lives through their different services in many important fields, such as healthcare, education, and smart homes. These devices have the advantages of connectivity and accessibility 24 h every day to collect real data instantly. However, concurrently, these devices together form an appealing environment for cybercriminals to launch attacks, specifically distributed denial-of-service (DDoS) attacks. Therefore, exploiting IoT devices to form a IoT botnet poses a threat that may affect precious resources. Commonly, a botnet can be identified as a collection of compromised devices recognised as bots operating malicious code and managed by an administrator called the botmaster [2,3,4]. These bots can propagate throughout networks by scanning for vulnerable devices and exploiting them in a process that aims to extend the botnets. Various types of malware have been issued and earmarked for IoT devices and aim to form IoT botnets. Some of the botnets noticed in IoT networks are Mirai, Bashlight, and Torii [5,6,7,8]. These IoT botnets have different versions, and recently, they have expanded their activities, as Security Intelligence reports that the activity of Mirai variants has roughly expanded and multiplied [9].

**Figure 1:**
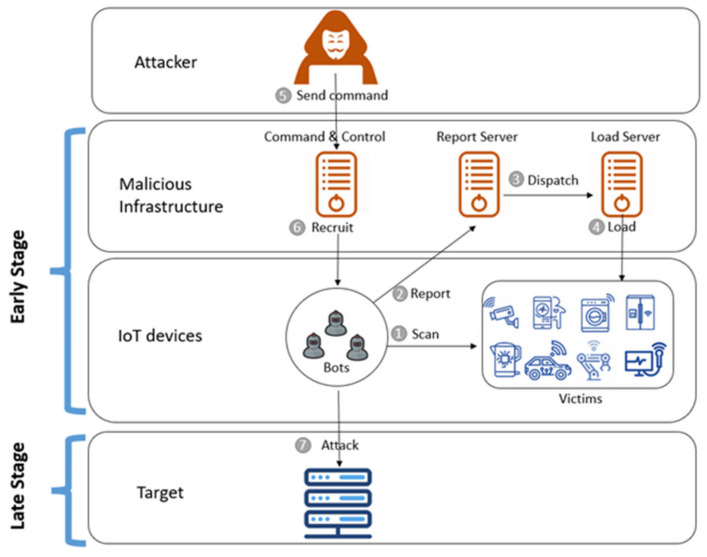
The IoT botnet formation stages [10].

IoT botnets carry out their activities in at least two main stages, the early stage and late stage (see Figure 1), and each stage has different malicious activities. The researchers in [10] explained these activities in detail. Generally, the two stages are as illustrated below.

- Early stage: In this stage, the attacker aims to weaponise IoT by scanning for new vulnerable IoT devices, such as devices with weak credentials or known vulnerabilities, which then download the bot, thereby exploiting these devices. Furthermore, the bot makes the necessary communication with the botmaster waiting for the attack command. At the same time, the bot scans for new vulnerable devices to be exploited with the aim to expand the botnet as much as possible.
- Late stage: In this stage, the attacker triggers a command to launch the attack by using the IoT botnet.

According to the above explanations of each stage, the detection of an IoT botnet in the early stage differs from detection in the late stage because each stage has different malicious activities. The detection in the early stage involves detecting the malicious scan for IoT devices, detecting the exploitation of vulnerable devices, and recruiting them by adding these devices to the botnet to be under the control of the attacker. On the other hand, detecting the botnet in the early stage concentrates on detecting the attack activity after initiating the attack command. The late-stage activities are not of interest in this research.

These unsafe devices accelerate and ease cybersecurity attacks, specifically when using a botnet. Moreover, restrictions on IoT device resources, such as limitations in power consumption and the central processing unit and memory, intensify this issue because they limit the security techniques that can be used to protect IoT devices.

In cybersecurity, artificial intelligence, machine learning, and deep learning models can be employed to create impressive tools to identify and then combat malicious behaviours. AI models and ML algorithms can analyse data, detect and realise sophisticated patterns within it, and foresee future effects depending on the data. The major feature is that the models and algorithms learn as they go, becoming more intelligent and more progressive, gaining the capability to detect the appointed cyberattacks and, at the same time, to predict how the forthcoming attacks might look. Therefore, machine learning and deep learning are robust tools to use in cybersecurity issues. The major trait of deep learning compared to classical machine learning approaches is its preferable performance in several situations, especially when learning from security datasets of considerable size. Deep learning fusion methods can be used to intelligently tackle various cybersecurity issues [11].

The convolutional neural network (CNN) [12] is a deep learning network architecture that learns directly from data, without the need for manual feature extraction. Although CNNs are most frequently applied to analyse visual imagery, these networks can also be used in the domain of cybersecurity to improve the accuracy of the detection of malicious behaviour. For example, the CNN model is utilised for intrusion detection and denial-of-service (DoS) attacks. Moreover, it is used in IoT network security [13] and malware detection [14].

A long short-term memory (LSTM) network uses special units that can handle the issue of the vanishing gradient. It has a ‘memory cell’ that can store data in memory for long periods. Numerous LSTM models have been used by researchers in the cybersecurity field for applications such as intrusion detection [15], phishing detection [16], and botnet detection [17].

### 1.1. The Need to Detect IoT Botnet in Early Stage

The IoT botnet threat is a challenge facing the Internet of Things (IoT) and requires effective methods and techniques for prevention. Numerous approaches could offer improvements in the detection of IoT botnets and enhance the whole security of IoT networks. In the recent literature on IoT, there is a shortage of in-depth studies on solutions for IoT botnet early-stage detection. Consequently, the research is somewhat immature and promising. The formation of a botnet has several stages; thus, the detection techniques should diverge based on the stages. Each stage reveals different actions; thus, a detailed analysis of the detection tactics in each phase is required. However, hitherto, there has not been enough research on IoT botnet detection with the early stage borne in mind. The late stage consists of attack activities that happen rapidly, so it is more logical to focus on the early stage, in which the botnet is formed and expands over a long period of time, which is a significant issue. Hence, we found the need for a detection method for IoT botnets that concentrates on the early stage. The proposed methodology improves the accuracy of the detection of IoT botnets in the propagation phase (early phase). The next subsection explains the research questions of this study and the related motivations.

### 1.2. Research Questions

In Table 1, the authors describe the research questions that were posed to effectively detect IoT botnets.

### 1.3. Contribution

This study adds to the body of knowledge in the area of IoT botnet detection with the following contributions:

- A technical experiment was conducted to investigate how IoT malware behaves and how it forms the IoT botnet.
- The Cross CNN_LSTM model was used to detect the IoT botnet in the early stage.
- A comparison of the evaluation of traditional ML classifiers with the proposed method was conducted.
- IoT botnet detection employing binary and multi-decision classes was implemented.
- The proposed methodology’s evaluation was compared with that of previous DL models and other baseline research.
- The proposed model significantly improved the IoT botnet detection ability.
- The proposed kill chain model focuses on detecting IoT botnets in the early stage.

The remaining parts of this article continue in the following manner: Section 2 thoroughly reviews the literature in the field of Internet of Things. The methodology of the study is described in Section 3. Section 4 addresses the key findings of the conducted experiments. Section 6 includes the limitations of the study. Section 6 concludes the study and points out directions for future work.

## 2. Literature Review

This section provides an intensive review of recent efforts in the area of IoT botnet detection and taxonomy. In addition, it recaps and assesses the current research articles.

Recently, various articles have surveyed the literature on IoT botnet detection. The authors in [10] presented a thorough analysis of experimental works related to the detection of IoT botnets. They provided a systematic literature review (SLR) by applying an effective method for assembling and critically examining research papers. This work focused on the detection methods used to detect IoT botnets, the botnet formation phases, and distinct malicious activity scenarios. The authors analysed the selected research and the associated key methods. They provided a classification for the detection methods based on the techniques used and studied the botnet phases during which detection is achieved. In addition, the authors analysed the existing research gaps and recommended future research directions. Another survey [18] studied the growth, detection, mitigation, and present trends within the field of botnet research. It classified botnet detection and mitigation and explained the existing challenges and trends to help discover enhancements for new botnet mitigation studies. In [19], the authors proposed a framework for future research on IoT botnets, which can be grouped into exploration, solution, or operation according to the stage and the aims of the research. This framework helps in supporting researchers to push their research from the initial exploration stage to an operational product that can execute the detection and mitigation of IoT botnets.

Machine learning and deep learning are good tools that have been used by researchers to detect botnets. The researchers in [20] proposed a hybrid deep learning (DL) model that combines bidirectional long short-term memory with a convolutional neural network (CNN) to predict DDoS attacks. They employed a feature selection method to obtain the most effective features in the used dataset. The results of the experiment showed that the proposed CNN-BI-LSTM realised an accuracy of up to 94.52%.

Similarly, various algorithms in machine learning and deep learning have been used to design models to detect an IoT botnet in different formation phases. In [21], the researchers proposed a framework for intrusion detection to distinguish malicious attacks using an enhanced model of deep reinforcement learning (DRL). They compared the performance of the proposed IDS framework to logistic regression and naive Bayes models and showed an experimental test accuracy of 96.99%. The authors of [22] used different machine learning algorithms to classify legitimate and malicious behaviours. They used random forest (RF), K-nearest neighbours (K-NN), decision tree (DT), and support vector machine (SVM). The used models obtained accuracies of 0.9532, 0.9025, and 0.9315 for RF, KNN, and DT, respectively, whereas SVM did not achieve good results. In [23], the researcher employed principal component analysis (PCA) to decrease the dimension of the data by generating a reduced number of new parameters with a naïve Bayes (NB) classifier algorithm that comprises two types of models, namely, Bernoulli and Gaussian. The results of the experiment in this research confirmed that the naïve Bayes classifier algorithm using PCA could achieve good results in the botnet classification. Applying the Gaussian model showed an accuracy of 97.71%, precision of 96.90%, and recall of 97.49%. In [24], the researchers conducted different experiments with different datasets and compared a set of machine learning and deep learning algorithms. These models were linear, K-nearest neighbour, naïve Bayes, decision tree, and random forest, which achieved accuracies of 86.8, 95.1, 87.6, 95.3, and 95.6, respectively. On the other hand, they conducted the same experiment using multilayer perceptron (MLPN) and long short-term memory (LSTM) and achieved accuracies of 89.1 and 87.6, respectively. In [25], the authors examined and compared three recurrent deep learning algorithms: FastGRNN, LSTM, and GRU. They used the three models to identify infected and soon-to-be-infected devices. The results of the experiments showed AUROCs between 98.8% and 99.7%.

The authors in [26] proposed a model integrating a word-embedding layer with a bidirectional long short-term memory recurrent neural network (BLSTM-RNN) to identify IoT botnets. The suggested model was compared with a unidirectional LSTM-RNN and achieved an accuracy of 99%. For the different attack vectors used by Mirai, the two models equally achieved high-level precision and minimal loss metrics.

In [27], the authors made use of machine learning and deep learning techniques for detection. They concentrated on botnets affecting different IoT devices and developed ML-based models for each type of device. They used an IoT dataset generated by adding botnet attacks (Bashlite and Mirai) to different kinds of IoT devices. They developed a botnet detection model for each device using numerous multiclass classification ML models and deep learning (DL) models. They achieved up to a 91% F1-score for the CNN model.

The authors in [28] suggested a honeypot-based method and utilised machine learning algorithms. The proposed solution captures attempts to download malware onto the IoT device. The gathered information was trained using the machine learning model. Utilizing the honeypot method to train the model was more efficient than using the limited known data, so unidentified variants of malware families with new features can also be used to train the model.

In [29], the researchers proposed a method to produce a printable string information graph (PSI) to indicate the connections, which was very beneficial for enhancing the recognition of IoT botnet malware. They employed the graphic convolution neural network classifier to distinguish malware without acquiring formerly selected features. The conclusion of the experiment revealed that the PSI graph CNN classifier attained 92% precision and a 94% F-measure.

The researchers in [30] suggested a method concentrating on obtaining fundamental features of IoT device traffic and used incremental statistics by employing the z-score technique to normalise the features. Then, they used the multivariate correlation analysis (MCA) algorithm based on triangle area maps (TAMs) to generate the dataset. They developed a convolutional neural network to train on the dataset and execute the detection phase. The experiment revealed that the suggested method attained 99.57% precision.

In [31], the authors proposed a model based on building a classifier for each IoT device separately; it focused on usage perspectives depending on core networks. They used a feature selection method to lower the number of attributes to facilitate the detection process. They proved that a multiclass classifier built on a shallow process, a decision tree, and fewer features could achieve very high precision rates from 94% to 98%.

The researchers in [32] established an agile detection system, namely, ConnSpoiler, that can precisely detect IoT botnets in a resource-limited manner. ConnSpoiler works by quickly classifying the flows of NXDomain queries to break the C&C link. The results demonstrated that ConnSpoiler had a 94% probability of identifying queries prior to their being sent to the C&C.

In [33], the authors presented a CNN-based deep learning model including a data-processing component and an eight-layer CNN. They segmented and normalised the energy utilisation data to help the CNN model to achieve better precision. The model classifies processed data into four categories, including the botnet class. They conducted a cross-device evaluation and leave-one-device-out and leave-one-botnet-out assessments on three conventional types of IoT devices. The assessment achieved an accuracy of 96.5%, cross-tests achieved 90% accuracy, and the leave-one-out examinations achieved more than 90% accuracy.

The article in [34] used machine learning methods to examine IoT botnets. The authors applied four ML algorithms using the USNW-NB15 dataset, i.e., DT, ARM, NB, and ANN. They assessed the accuracy and false alarm rate. The outcomes revealed that DT enhanced the detection process with an accuracy of 93%.

In [35], the authors proposed a method to identify IoT botnet actions by utilizing the grey wolf optimisation (GWO) algorithm to improve the hyperparameters of the support vector machine and ranked features. The experimental outcomes on a subsection of the N-BaIoT dataset indicated that GWO enhanced the classification process of the one-class support vector machine. It reached an accuracy between 96–99%.

**Figure 2:**
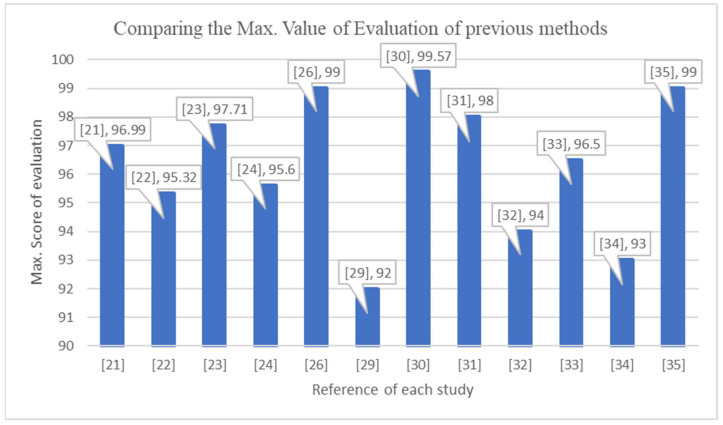
The max. value of evaluation of each method that has been used in state-of-the-art studies to detect IoT botnet in early stage and late stage.

Despite the importance and effectiveness of early-stage detection in stopping the botnet before it starts the attack, not enough work has been performed in this area. Figure 2 demonstrates the max. value of evaluation of each method that was used in state-of-the-art studies to detect IoT botnets in the early stage and late stage. It is clear that previously used methods to detect a botnet in the early stage did not achieve a level of accuracy as well as others in the late stage. Table 2 is divided into two parts: the first demonstrates the methods that were used in state-of-the-art studies to detect the IoT botnet in the late stage, and the second demonstrates the methods that detect the botnet in the early stage. It is obvious that few of these works concentrated on early-stage detection, and their achieved accuracy still needs to be improved for effective detection. On the other hand, it is clear that using deep learning models has achieved promising accuracy. Therefore, the proposed model in this research paper concentrates on detecting IoT botnets in the early stage and improving the accuracy by using a deep learning algorithm.

**Figure 3:**
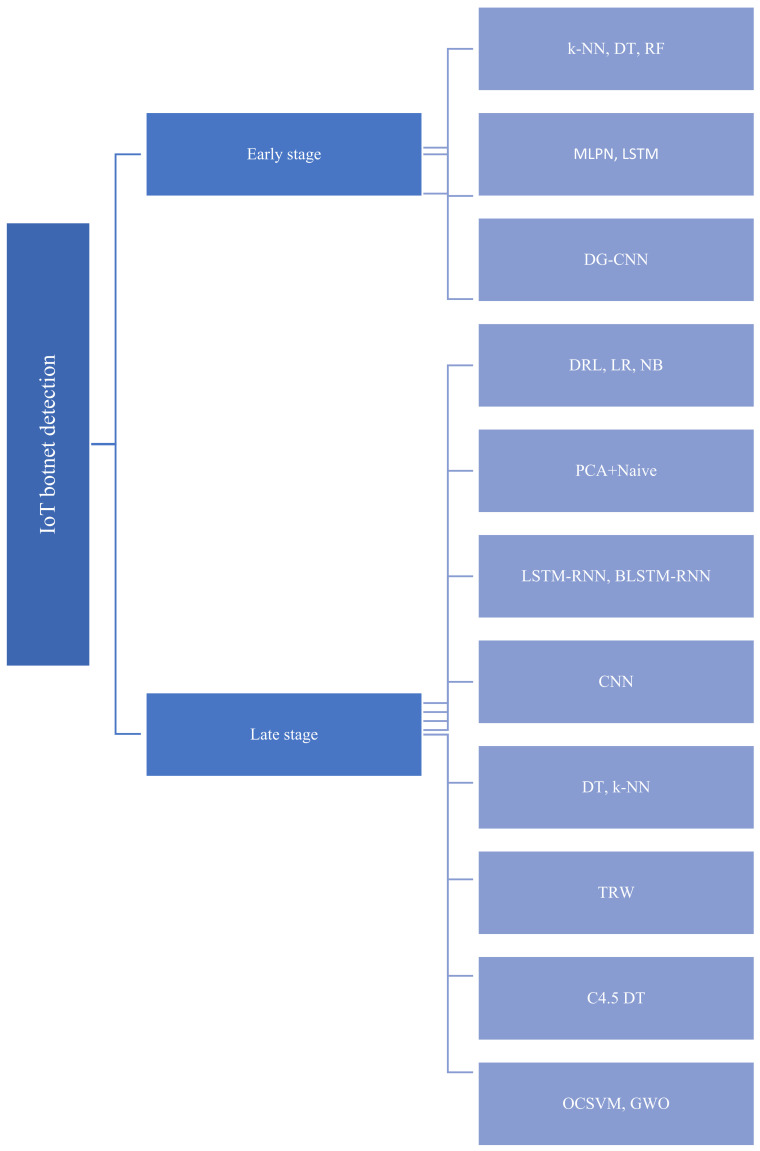
Taxonomy of state-of-the-art methods used to detect IoT botnets in early stage and late stage.

The taxonomy in Figure 3 classifies state-of-the-art methods that have been proposed to detect IoT botnets in the early stage and late stage.

## 3. Materials and Methods

**Figure 4:**
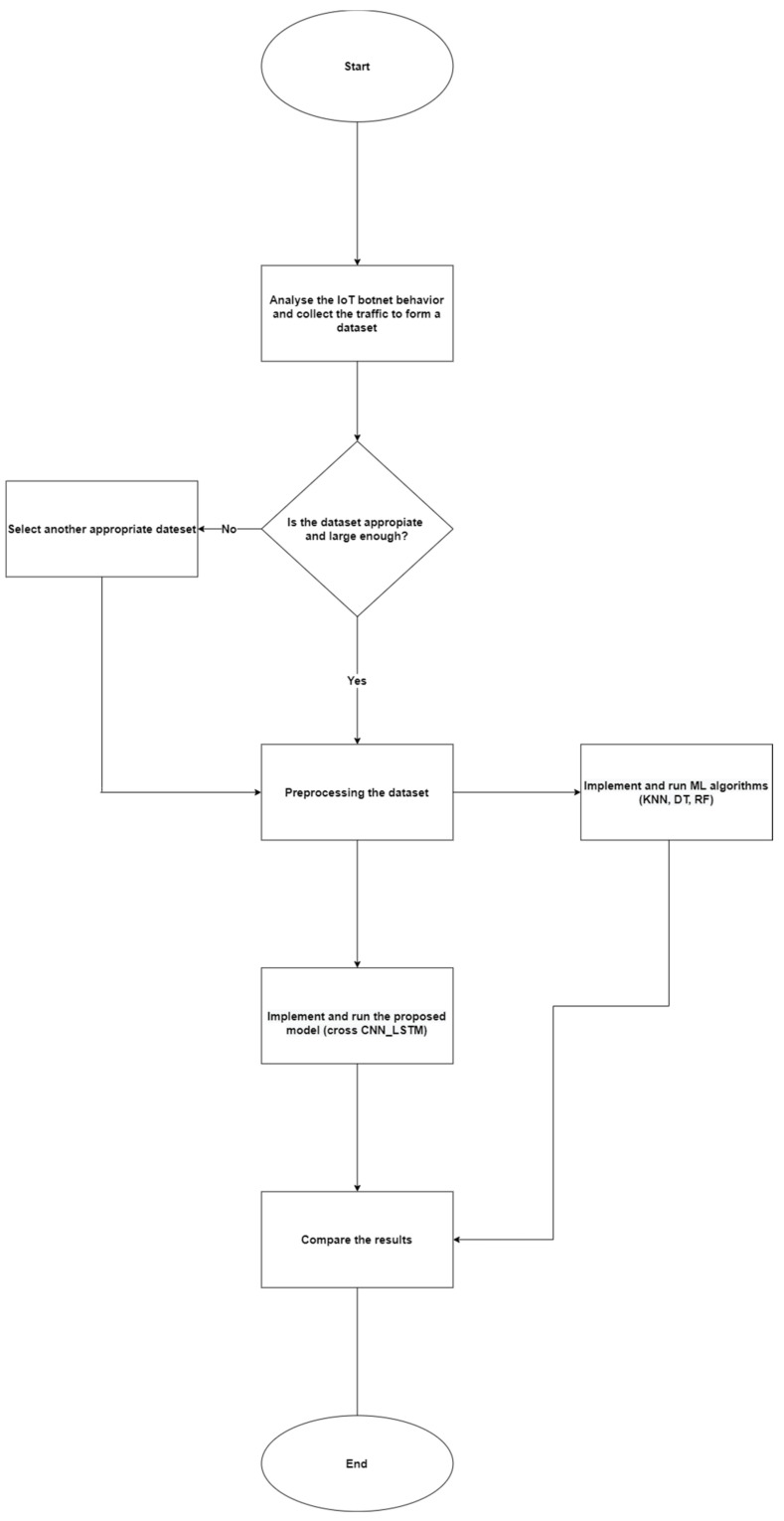
A comprehensive scheme of the used methodology.

This section consists of two parts. The first part explains our prototype, which is used to investigate and analyse the IoT botnet and malware behaviours when forming the botnet. The second part is about adapting the convolutional neural network and long short-term memory in the proposed classification model. The following subsections discuss the whole methodology of this research: dataset selection, feature selection, dataset sampling, data preprocessing, architecture design, and experimental setup. Figure 4 gives a comprehensive scheme of the used methodology.

### 3.1. A Prototype for Analysis of IoT Botnet Propagation

This subsection concerns finding the answer to RQ1. It is necessary to understand and analyse the behaviour of the IoT botnet before starting to design a detection model. Therefore, this study provides a prototype that investigates the behaviour of the IoT malware and how it starts to form the botnet in the IoT network. Through the following experiment, we investigated the early stages of Mirai, as it is the most famous IoT malware that forms the largest IoT botnet.

#### 3.1.1. Testbed Environment

In this research, the testbed environment consists of one physical machine with virtual machines (VMs). This research used VMs because they afford an efficient and safe environment to perform an analysis of the botnet and to study its behaviour; at the same time, it is a flexible, adaptable means to deploy a testbed. On the other hand, if the testbed depends only on a physical machine to analyse the botnet, the cost of the experiment will be very high, so using virtual machines reduces the cost and affords the ability to reset the physical machine to the initial status if the virtual machines are contaminated with malware. In this way, we can repeat the experiment multiple times and acquire accurate results in a reliable manner.

#### 3.1.2. Testbed Components

**Figure 5:**
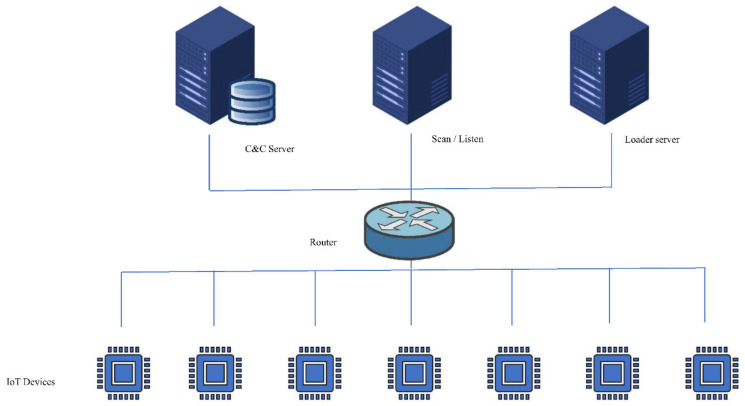
The architecture of the used testbed to simulate and analyse the behaviour of IoT malware.

In this subsection, we explain the components of the testbed that was used for the experiment in this research. Figure 5 shows the structure of the testbed and the components. This testbed consists of one physical machine on which we installed several virtual machines: one for the C and C server, which contains a database, the second for the scan/listen server, and the third for the loader server. For the IoT device, there are seven virtual machines, each of them representing a different IoT device. The research used a packet sniffer tool to sniff the traffic and analyse the packets.

#### 3.1.3. The Experiment

The main goal of this experiment was to analyse the IoT botnet malware and study its behaviour by monitoring and collecting traffic packets. In this experiment, we focused on studying IoT botnet propagation, so we concentrated on scanning, brute-forcing, downloading, and installing the malware binaries on the IoT devices.

**Figure 6:**
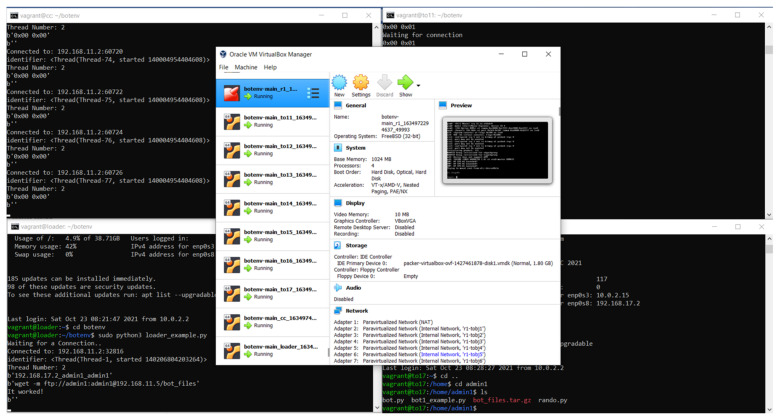
The deployed testbed environment.

Afterward, we performed the necessary configuration for VirtualBox [36] and Vagrant [37], and we downloaded the Mirai botnet source code, which is available through different project sources [38,39,40]. Then, we implemented the testbed, deployed and started all of the virtual machines in the environment, and operated the needed commands to monitor the traffic. We used the built-in capability of VirtualBox to collect the traffic and create pcap files by using VboxManage [41]. As a result, PCAP files were stored for analysis. Figure 6 shows the deployed environment.

**Figure 7:**
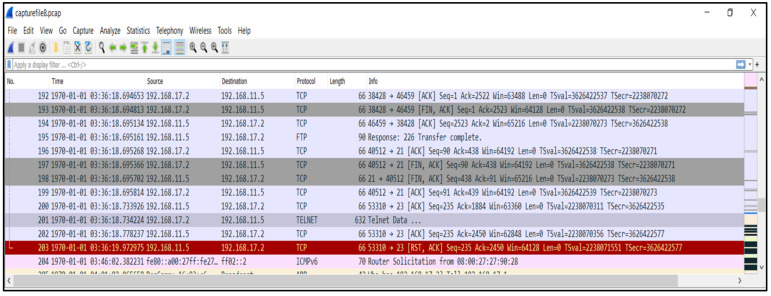
Utilisation of Wireshark to analyse the pcap files and follow the network packets.

**Figure 8:**
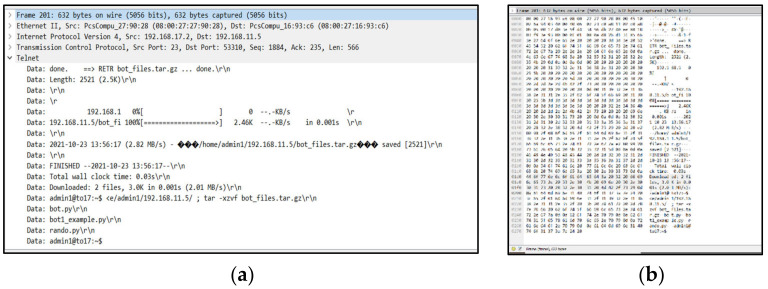
The investigation processes of the IoT botnet in the early stage: (**a**) focus on packet details; (**b**) focus on the hexdump of the packet.

After deploying the testbed environment and collecting the traffic, we utilised Wireshark [42] to analyse the pcap files and follow the network packets, as shown in Figure 7. Furthermore, we analysed the traffic and followed the communications between different IP addresses to instigate the IoT botnet in the early stage, including the infection process and the propagation process through the IoT devices. Figure 8 shows these investigation processes.

According to the above experiment, we can conclude that we could follow and analyse all steps in which Mirai acts to form the botnet, such as the scanning of vulnerable devices, communications between bots and C&C, and infection of virtual devices. This helps us to achieve a better understanding of the IoT malware behaviours and answer the first research question.

In this experiment, we tried to form a dataset to be used in the following steps of our methodology and to be employed in training the proposed model, but we faced the challenges that the generated dataset size was small and the limitation of using real IoT devices in the experiment. On the other hand, we found that there were different state-of-the-art IoT datasets that we could utilise in our models and received the benefit of comparing our model to other models that used the same dataset. The next sections explain this issue in detail. Thus, in the second part of the methodology, we explain our procedure and criteria for choosing the appropriate dataset.

### 3.2. The Proposed Model

This section starts by describing the selection of the appropriate dataset, sampling the dataset, and preprocessing it, and then it demonstrates the implementation of the ML models and the implementation of the proposed model to answer the second research question, RQ2.

#### 3.2.1. Dataset Selection

The quality and the size of the dataset significantly impact the performance of deep learning models. Unfortunately, as noted in Section 2, some of the researchers in IoT botnet detection use general datasets such as UNSW-NB15 [43], which may result in inaccurate models because IoT and associated malware behave differently from general-purpose computers and their malware. As a result, the research on IoT botnet detection suffers from a lack of benchmark datasets; however, efforts to build and publish a realistic IoT dataset to address this issue have recently generated IoT-based datasets, despite some shortcomings such as the imbalance problem, as in Bot-IoT [44], which may affect the performance of the proposed model. Therefore, this study followed specific criteria to select the dataset, as follows:

- The dataset should be generated using different types of IoT devices.
- More than one IoT malware should be used.
- A real IoT botnet binary code should be used to formulate the botnet.
- The dataset should focus on the early stages of deploying the IoT botnet, as explained in this section.

Based on the above criteria and as discussed in this section, the MedBIoT [22] dataset fills the gap in terms of the lack of IoT datasets generated in IoT botnet detection. It was generated using a medium-sized network of IoT devices consisting of 83 IoT devices. These devices are a combination of physical and emulated IoT devices. It provides real network data by deploying actual malware (Mirai, Bashlite, and Torii). This dataset focuses on the propagation stage (spreading and communication). The dataset consists of 23,340,359 network packets divided into different classes, as explained in Table 3.

#### 3.2.2. Feature Extraction

**Figure 9:**
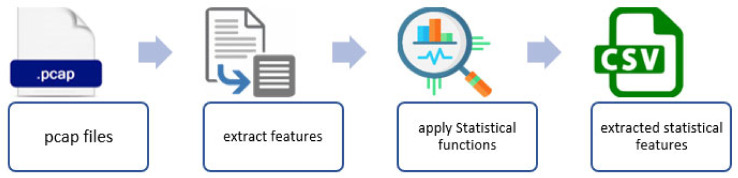
Feature selection and extraction.

The selected dataset (MedBIoT) provides two kinds of data: raw and structured data. The bulk structured data used for the purpose of this study were obtained from pcap files, and the statistical features were extracted using Splunk [45]. The total number of extracted features is 23, and they were selected according to five different time windows for the recent period (100 ms, 500 ms, 1.5 s, 10 s, and 1 min). Table 4 shows a description of these features. The features are divided into four types, which summarise all of the traffic between host and protocol communications. Type 1 refers to traffic produced by the same IP, Type 2 refers to traffic produced by the same IP and the same MAC, Type 3 refers to traffic between the same source and destination IP address, and finally, Type 4 refers to traffic between the same source and destination TCP/UDP. Figure 9 shows the process of feature selection and extraction from the pcap files.

#### 3.2.3. Dataset Sampling

As seen in Table 4, MedBIoT is a large imbalanced dataset, so the researcher used an undersampling technique to provide a balanced sample of the dataset; Table 5 demonstrates the dataset after undersampling. The researcher split the dataset into eight classes, legitimate and malicious (communication and spread) for each of the three malware types. As a result, the total number of instances is approximately 1,000,000 instances for the eight different classes.

**Figure 10:**
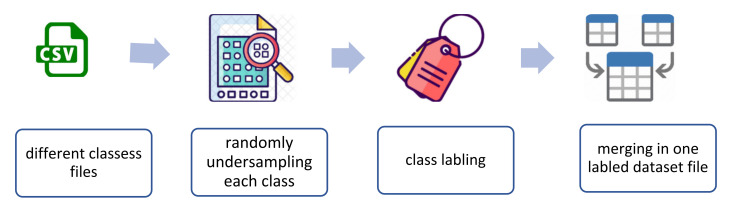
The process of undersampling and formulating the used balanced dataset.

The used undersampling technique uses random sampling with a specific fraction to obtain the desired number of instances depending on the size of the records in each class in the dataset. Then, all classes are labelled and gathered in one CSV file, as shown in Figure 10.

#### 3.2.4. Dataset Preprocessing

The dataset preprocessing process contains three steps: shuffling, normalisation, and splitting. Before the dataset is trained, the records of the dataset should be shuffled to ensure that the model will generalise well. In this step, the researcher applies a permutation method. After that, in the normalisation step, all columns are normalised by standardizing all values to be between 0 and 1.

To estimate the performance of the deep learning algorithms for predictive modelling problems, the dataset should be split into training, validation, and test datasets. For this purpose, the researcher used the train_test_split method [46] to split the dataset into training, validation, and test data using a ratio of 70:20:10.

#### 3.2.5. Implementation of Baseline Machine Learning Models

To test our dataset, first, we tried to read the dataset and run different baseline machine learning models to gain insight into the applicability of the prepared dataset. We used algorithms on the same dataset for the proposed model. First, we implemented the three baseline machine learning algorithms K-nearest neighbours, decision tree, and random forest.

The K-nearest neighbour algorithm (KNN) [47] is one of the simple, efficient, and straightforward-to-apply supervised machine learning algorithms. It is usually used in classification and regression scenarios. It depends on similarity scores (e.g., distance function) such as Euclidean distance (see Formula (1)).

(1) $$\sqrt{\overset{k}{\underset{i=1}{\sum }}{\left(x_{i}-y_{i}\right)}^{2}}$$

A decision tree algorithm (DT) [48] supports the decisions and the potential outcome. It has a hierarchical structure and tree structure employing acyclic directed graphs. It begins with a root node that splits into two branches, forming the next level of nodes, which continue splitting until reaching leaf nodes using the entropy coefficient, which takes a value between 0 and 1 (see Formula (2)) in each split.

(2) $$E\left(S\right)=\overset{c}{\underset{i=1}{\sum }}-p_{i}log_{2}p_{i\;}$$

where *pi* is simply the Bayesian probability of class *i* of the dataset.

The random forest algorithm (RF) [49] is also a supervised machine learning algorithm. It is used widely in classification and regression problems. It consists of many decision trees and makes the prediction from each tree. It foresees the last result based on the majority votes of all predictions.

The results of this experiment are shown in Table 6.

Section 4 demonstrates a comparison between these results and the results of the proposed model.

#### 3.2.6. Architecture Design of the Proposed Model

**Figure 11:**
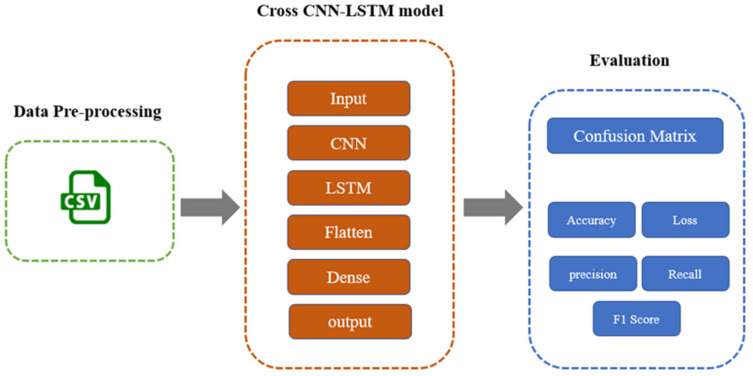
The proposed architecture design.

The proposed hybrid model consists of different layers: an input layer, CNN layer, LSTM layer, flatten layer, dense layer, and output layer, as described in Figure 11. Once the preprocessing process is finished, the resulting vector is used as an input to the model. Algorithm 1 demonstrates the pseudocode of the model. In the first layer, CNN has 128, 64 neurons as input, and the second layer (LSTM) has 32, 16 neurons. The dense layer has 128, 64 neurons. These two layers are used in combination in the model because they produce a high-accuracy model. In the flatten layer, the vector is flattened or reshaped into a one-dimensional vector to be used in the dense layer. The model has a dropout layer with a rate of 0.2 to avoid model overfitting, which is implemented by randomly dropping some neurons from the last layer. In the dense layer with the ReLU activation method, the output is generated. For compiling the model, the researcher used a categorical cross-entropy loss function for multiclass classification and binary categorical cross-entropy for binary classification. Moreover, the researcher used an Adam optimiser and ReduceLROnPlateau function for tuning the learning rate and then trained the model with 50 epochs and early stopping after 10 epochs when there was no improvement in the loss. All of the hyperparameters are explained in Table 7.
**Algorithm 1** Algorithm for the proposed model Input: Preprocessed dataOutput: Accuracy, loss, precision, recall, F1-score1:  Standardise (Preprocessed_data)2:  Shuffle (Preprocessed_data))3:  Split (Preprocessed_data) based on 70:10:20 (training_data, validating_data, test_data)4:  Apply CNN layer5:  Apply LSTM layer6:  Flatten7:  Apply Dense8:  Use Adam optimiser9:  Use a categorical cross-entropy as loss function10: for (epoch = 1; epoch < 50; epoch++) do11:    evaluate loss, validation loss12:   evaluate accuracy, validation accuracy13: end for14: Use testing data to calculate precision, recall, F1-score15: Calculate loss, accuracy

The authors can provide the implementation and the used dataset upon request to encourage researchers to repeat the experiment and use different hyperparameters for tuning.

#### 3.2.7. Experimental Setup

The proposed model in this research was written in Python language version 3.8.5, which is powerful in data science and has a collection of useful libraries, such as Pandas, NumPy, matplotlib, sklearn, and others [50]. In addition, Python is listed as the top programming language for embedding systems such as IoT devices [51]. The experimental environment consisted of a laptop with AMD Razon 7, 2900 Mhz, 8 cores, 16 logical cores with 16 GB memory, and Nvidia Getforce GTX 1660 Ti. Different packages were used, such as Anaconda [52], Tensorflow [53], and Keras [54].

## 4. Results and Discussion

### 4.1. Experimental Results

The model is evaluated using a confusion matrix [55], which consists of four evaluation metrics (see Table 8) as follows:

- True Positive (TP): where the proposed model correctly predicts the positive class;
- True Negative (TN): where the proposed model correctly predicts the negative class;
- False Positive (FP): where the proposed model incorrectly predicts the positive class;
- False Negative (FN): where the proposed model incorrectly predicts the negative class.

Based on these metrics, the evaluation method calculates the precision, recall, and F1-score as illustrated below:

- Precision: the proportion of the true positive to all positive:

P = TP/TP + FP

- Recall: the proportion of the true positive to all relevant elements:

R = TP/TP + FN

- F1-Score: a combination of precision and recall:

F1 = 2. P.R/P + R or F1 = TP/TP + 1/2 (FP + FN)

The following tables show the results and measurements for precision, recall, and F1-score for the binary and multiclass classifications. In the binary classification, we classified the traffic as malicious and legitimate, as explained in Table 9. On the other hand, we performed two multiclass classifications: one with three classes, which are demonstrated in Table 10 and are communication, spread, and legitimate, and one with four classes to distinguish between Mirai, Bashlite, and Torii, as explained in Table 11.

As explained before, CNN can considerably decrease the number of parameters, and this enhances the efficiency of model learning. Moreover, LSTM has its own memory and can make relatively accurate classifications. Therefore, the Cross CNN_LSTM architecture uses CNN layers to perform the feature extraction on input data, and it is combined with LSTMs to support the prediction. From the previous tables, we can notice that the results of the proposed Cross CNN_LSTM model show a good detection rate. It achieved an accuracy score between 99.2% and 99.7% in general. The binary classification for the two classes, legitimate and malicious, had 99.23% accuracy. The results of the three-class multiclassification with the classes legitimate, spread, and communications show 99.44. Finally, the four-class multiclassification with the classes legitimate, Mirai, Bashlite, and Torii had an accuracy of 99.7% and averages of 99.68%, 99.67%, and 99.67% for recall, F1-score, and precision, respectively.

In this subsection, we demonstrate how the new proposed model employs deep learning in detecting the propagation of the botnet in IoT networks, and this answers the second research question, RQ2.

### 4.2. Comparison against State-of-the-Art

This section conducts a comparison between the proposed model and benchmark studies. However, this kind of comparison is challenging due to a set of restrictions. Such models are assessed on different datasets or different sizes of instances and have been tested in different environments. Moreover, the contributing researchers presented their models in related studies without enough details about their experiments, which could make the comparisons unrealistic.

Keeping in mind the mentioned challenges, in this work, for the sake of comparisons, we followed the following strategy (multiclass comparisons):

- Compare the proposed Cross CNN_LSTM to the set of our implemented baseline machine learning algorithms, KNN, DT, and RF. See Section 3.
- Compare the proposed Cross CNN_LSTM to the machine learning models KNN, DT, and RF that were implemented for the early stage in [22].
- Compare the proposed Cross CNN_LSTM to the deep learning model presented in state-of-the-art works and focus on early-stage detection; however, they used different datasets, such as CNN and DG-CNN, in [29,33], respectively.

### 4.3. Discussion

This subsection answers research questions RQ3 and RQ4. As we see in Table 12, the comparison includes different types of studies according to the comparison policies. Notice that the authors use an average score of the four different classes (Mirai, Bashlite, Torii, and Benign) of the measurements of F1-score, precision, and recall, and this is to use only one number for the sake of comparison to the other works. Generally, we can see that studies that used deep learning algorithms outperformed the other studies that used machine learning algorithms. The studies in [22,23,24] used the same dataset with diverse machine learning algorithms. For the studies in [29,33], they used different datasets. Unfortunately, some of the studies [29,33] did not provide all of the metrics, so some of the scores are missing. According to the conducted experiments, the results show that the suggested model is accurate and outperforms the state-of-the-art methods, and it achieves 99.66 accuracy. Moreover, the authors measured the training time and the detection time, and the results show that the training time of the model was 7 h, 1 min, and 28 s; on the other hand, the detection time was 36 s. The authors believe that the model can achieve better training time if a feature reduction method is used. In the last section of this study, we highlight potential future works.

## 5. IoT Botnet Kill Chain Model

With the growth of the number of connected devices, at the same time, linked threats also rise. Understanding the evolution of malware that aims to infect IoT devices is essential to implementing efficient countermeasures and protection. There are two methods that can be used to help protect IoT networks from attacks, namely, the MITRE ATT&CK framework [56] and the Lockheed Martin Cyber Kill Chain model [57]. This section develops an IoT botnet early-stage detection-based framework by mapping the MITRE ATT&CK model to understand adversarial tactics and techniques. Moreover, an IoT botnet kill chain model is implemented by applying a risk strategy for earlier-stage detection.

The MITRE ATT&CK model is a well-known, internationally open knowledge base of adversary tactics and techniques based on real-world observations. This knowledge base is utilised as a groundwork for the development of specialised threat models and methods.

In this study, first, we projected the Mitre Att&ck framework on the IoT botnet early-stage detection framework. There are many tactics used by IoT malware, including Reconnaissance, Initial Access, Credential Access, Lateral Movement, Defence Evasion, Execution, Persistence, and Discovery. Moreover, these malware types use different related techniques for each tactic, as explained in Table 13.

On the other hand, the Lockheed Martin Cyber Kill Chain framework is composed of the Intelligence Driven Defense model for the classification and avoidance of cyber intrusion endeavour. The model recognizes what the adversaries must carry out in order to accomplish their goals.

**Figure 12:**
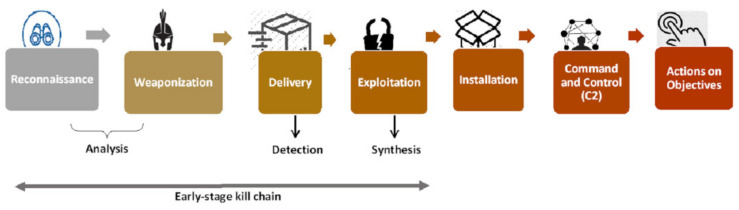
IoT botnet kill chain model.

We provide a systematic process for an IoT botnet kill chain aligned with the Lockheed framework. We aim to study the tactics used by cyber adversaries so that we can decrease the adversary’s opportunity to form the IoT botnet and prevent it in the early stage. The phases for the early-stage detection of the IoT botnet are described in Figure 12 and explain several protective countermeasures that can break down this kill chain. The following steps explain the Lockheed Martin Kill Chain framework:

1. Reconnaissance;
2. Weaponisation;
3. Delivery;
4. Exploitation;
5. Installation;
6. Command and Control (C&C);
7. Actions on Objectives.

From Figure 12, we can notice that there are three important countermeasures that should be taken into consideration for early-stage detection of the IoT botnet:

1. Analysis at time of weaponisation;
2. Detection during delivery;
3. Synthesis between various exploitations.

**Figure 13:**
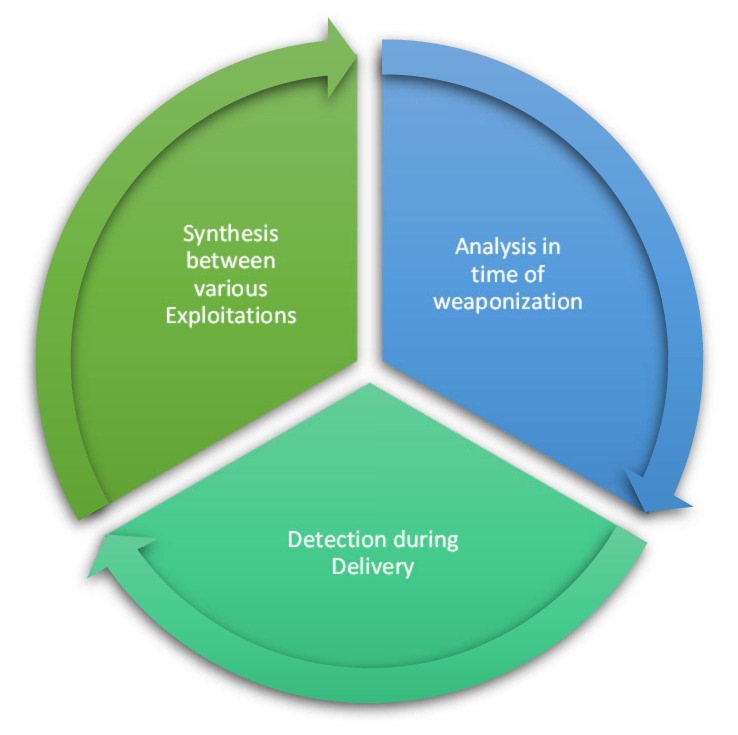
IoT botnet kill chain model for early stage.

Figure 13 explains the three countermeasures of the life cycle for the IoT botnet kill chain in early-stage detection to avoid infection with and spreading of the IoT botnet and, at the same time, prevent the attackers from extending the botnet. The three countermeasures are explained in detail as follows:

- Analysis at time of weaponisation: This countermeasure can be covered by different techniques. The traffic should be analysed, and the investigation should be conducted to find any scanning activities. Scans can be performed manually or automatically to detect any activities of gathering host information and communications to send to C&C or any brute-forcing, remote access, system restarts, loss of credentials, or other failures.
- Detection during delivery: This countermeasure can be implemented by investigating the existence of any malicious binaries that can be downloaded on IoT devices and removing them periodically.
- Synthesis between various exploitations: All unsuccessful attempts to brute force credentials and any downloaded file attempts should be taken into consideration because the attacker may repeat these attempts through the network and execute successful attempts.

## 6. Limitations of the Study

In this study, we faced different challenges, so this study has the following limitations that should be overcome for better development of the proposed methodology:

- In the developed prototype, we could not use physical IoT devices, so we implemented a virtual environment, and we repeated the experiment many times with different changes for a better understanding of the IoT botnet behaviour. This is because the cost would have been too high if we used real physical IoT devices since repeating the experiment may require replacing the affected device with a new one every time that we repeat the experiment.
- Deep learning does not have a technique to randomly subsample the output and decrease the capacity or diminish the network during the training phase, so the model does not have an implanted technique to prevent overfitting that may occur when training the model.

## 7. Conclusions and Future Work

Increasingly, IoT botnets are using techniques that make them more effective and more difficult to detect. Consequently, it has become one of the cybersecurity concerns. This research paper reviews state-of-the-art studies on IoT botnet detection and offers a brief description of each study, with the goal of enriching the knowledge of different methodologies to detect IoT botnets and providing a taxonomy of the articles depending on the botnet stage that they studied, namely, the early stage or late stage. The authors provide a prototype that was subjected to technical empirical experiments to investigate the behaviour of IoT malware, which provides a good understanding of the early stage of forming the IoT botnet and answers RQ1. Most of the previous studies focused on the late stage, which happens rapidly, whereas it is more logical to focus on the early stages, in which the botnet is formed and expands over a long period of time, which is a significant issue in detecting IoT botnets and preventing DDoS attacks. Moreover, the authors developed multiclass classification methods using a fusion deep learning model, namely, Cross CNN_LSTM, and employed a real IoT dataset for the early stage of the IoT botnet to answer RQ2. Various experiment attempts were carried out, and a comparison was conducted by comparing the proposed methods to different previous works that utilised baseline machine learning methods and some deep learning methods. The results of the experiments answer RQ3 and RQ4. They show that our proposed method outperformed the other methods in terms of different evaluation metrics: precision, recall, accuracy, and F1-score. We confirmed that the proposed Cross CNN_LSTM model outperformed the other models by increasing accuracy, achieving 99.66, 99.68, 99.67, and 99.67 accuracy, recall, F1-score, and precision, respectively. Consequently, a framework for IoT botnet early-stage detection based on MITRE ATT&CK was developed, and an IoT botnet kill chain model based on the Lockheed Martin model was implemented by applying a risk strategy for earlier-stage detection.

The area of research on IoT botnet detection is a fertile field, specifically when using deep learning algorithms. For future work, we intend to test our proposed models with different IoT datasets to evaluate our model. We also plan to expand our prototype and enhance the experiment to generate a dataset by capturing IoT network traffic across Internet of Things devices. We will assess the performance of our model in terms of calculating and enhancing training and detection time. We will put more effort into examining dimension reduction and efficient feature selection methods, which may enhance the performance of the model. Additionally, we will examine and compare more deep learning algorithms, such as autoencoder, which attains good accuracy, as well as GRU, to the proposed Cross CNN_LSTM model. Finally, the proposed model can be integrated with another one that concentrates on DDoS attack detection.

## Figures and Tables

**Table 1:** The research questions.

Research Question	Motivation
*RQ1. How does IoT malware behave in the IoT network to form a botnet?*	Investigate how IoT malware such as Mirai starts to form a botnet in the IoT network with a concentration on the early stages in formulating the botnet.
*RQ2.* How can the Cross CNN_LSTM Deep Learning model identify IoT botnet detection based on a benchmark dataset?	Examine the proposed cross deep neural network model CNN_LSTM and employ it to detect botnets using a benchmark dataset.
*RQ3.* How can we compare the proposed Cross CNN_LSTM Deep Learning model to traditional ML techniques?	Investigate conventional machine learning approaches such as random forests (RF), k-nearest neighbour algorithm (k-NN), and support vector machine (SVM), along with a variety of evaluation metrics such as accuracy, precision, recall, and F1-score.
*RQ4. How do we compare the* proposed *technique’s accuracy in detecting IoT botnets employing a benchmark dataset to baseline and other deep learning approaches?*	Investigate state-of-the-art approaches that use deep learning with a variety of evaluation metrics, such as accuracy, precision, recall, and F1-score.

**Table 2:** The methods that were used in state-of-the-art studies to detect IoT botnet in early stage and late stage.

Authors	Year of Publication	Stage	Method	Maximum Score of Evaluation	Reference
*Gupta, Govind P.*	2022	Late	DRL, LR, NB	96.99%.	[21]
*Aprianti et al.*	2021	Late	PCA+Naive	97.71%	[23]
*McDermott et al.*	2018	Late	LSTM-RNN BLSTM-RNN	99%	[26]
Liu et al.	2019	Late	CNN	99.57%	[30]
*Bahşi et al.*	2018	Late	DT, k-NN	98%	[31]
*Yin et al.*	2019	Late	TRW	94%	[32]
*Jung et al.*	2020	Late	CNN	96.5%	[33]
*Koroniotis et al.*	2017	Late	C4.5 DT	93%	[34]
*Al Shorman et al.*	2020	Late	OCSVM, GWO	99%	[35]
*Guerra-Manzanares et al.*	2020	Early	k-NN, DT, RF	95%	[22]
*Gandhi et al.*	2021	Early	RF, MLPN, LSTM	95%	[24]
*Nguyen et al.*	2018	Early	DG-CNN	92%	[29]
*Our proposed model*	-	Early	CNN+LSTM	-	-

**Table 3:** Number of packets in dataset according to malware type.

Traffic Type	Number of Devices	Number of Packets
BashLite	40	4,143,276
Mirai	25	842,674
Torii	12	319,139
Benign	83	12,540,478
Sum	160	17,845,567

**Table 4:** Features in the dataset.

	Types	Features	Number of Features
1	Host MAC and IP	Packet count, mean, and variance	3
2	Channel	Packet count, mean, variance, magnitude, radius, covariance, and correlation	7
3	Network Jitter	Packet count, mean, and variance of packet jitter in channel	3
4	Socket	Packet count, mean, variance, magnitude, radius, covariance, and correlation	7

**Table 5:** The used dataset after undersampling depending on traffic class.

Malware	Type of Class	Class	Number of Instances
Mirai	Legitimate	mirai_leg	167,000
	Communication	mirai_mal_CC	100,000
	Spread	mirai_mal_spread	100,000
Bashlite	Legitimate	bashlite_leg	167,000
	Communication	bashlite_mal_CC	100,000
	Spread	bashlite_mal_spread	100,000
Torii	Legitimate	torii_leg	167,000
	Spread and communication	torii_mal_all	100,000

**Table 6:** Measurement results for ML classifiers.

Model	Accuracy	Recall	F1-Score	Precision
KNN	90.0	91.8	92.1	92.0
DT	91.0	93.5	93.625	93.5
RF	94.0	95.125	95.375	95.5

**Table 7:** Hyperparameters of the model and their values.

Hyperparameter	Value
CNN units	128, 64
LSTM units	64, 32
Epochs	50
Early stopping	10
Starting learning rate	0.001
Activation	ReLU
Loss	Categorical cross-entropy, binary categorical cross-entropy
Optimiser	Adam

**Table 8:** Confusion Matrix.

	Predicted Class	
**Actual Class**	**Positive**	**Negative**
Positive	TP	FN
Negative	FP	TN

**Table 9:** Measurement results for 2 classes.

Model	Classes	Accuracy	Recall	F1-Score	Precision
Binary classification	Legitimate	99.23	99.17	99.23	99.30
	Malicious	99.23	99.30	99.23	99.17

**Table 10:** Measurement results for 3 classes.

Model	Classes	Accuracy	Recall	F1-Score	Precision
Multiclassification	Legitimate	99.44	99.49	99.46	99.43
	Spread	99.44	99.50	99.52	99.53
	CC	99.44	99.28	99.32	99.35

**Table 11:** Measurement results for 4 classes.

Model	Classes	Accuracy	Recall	F1-Score	Precision
Multiclassification	Legitimate	99.66	99.70	99.70	99.70
	Mirai	99.66	99.15	99.19	99.23
	Bashlite	99.66	99.92	99.92	99.92
	Torii	99.66	99.95	99.88	99.81

**Table 12:** Results of the conducted comparisons (N/A = not available).

Type of Model	Dataset	Ref.	Model	Accuracy	Recall	F1-Score	Precision
Machine Learning Models	MedBIoT dataset	Our ML Models	KNN	90.0	91.8	92.1	92.0
			DT	91.0	93.5	93.625	93.5
			RF	94.0	95.125	95.375	95.5
		[22]	KNN	87.06	87.06	85.05	88.49
			DT	95.16	95.84	95.16	94.99
			RF	97.66	98.24	97.66	96.57
Deep Learning Models	Other	[29]	DG-CNN	92	N/A	94	N/A
		[33]	CNN	96.5	N/A	N/A	N/A
	Our Cross CNN_LSTM		CNN + LSTM	99.66	99.68	99.67	99.67

**Table 13:** IoT botnet early-stage detection framework based on MITRE ATT&CK.

Tactics	Reconnaissance	Initial Access	Credential Access	Lateral Movement	Defence Evasion	Execution	Persistence	Discovery
Related Techniques	Active Scanning	External Remote Services	Brute Force: Password Guessing	Exploitation of Remote Services	Indicator Removal on Host: File Deletion	Command and Scripting Interpreter	Pre-OS Boot: System Firmware	Process Discovery
	Vulnerability Scanning				Environment Keying			

## Data Availability

Not applicable.
